# Field spectroscopy data from non-arable, grass-dominated objects in an intensively used agricultural landscape in East Anglia, UK

**DOI:** 10.1016/j.dib.2019.104888

**Published:** 2019-11-28

**Authors:** Ute Bradter, Jerome O'Connell, William E. Kunin, Caroline W.H. Boffey, Richard J. Ellis, Tim G. Benton

**Affiliations:** a University of Leeds, School of Biology, Leeds, LS2 9JT, UK; b Norwegian Institute for Nature Research, P.O. Box 5685 Torgard, 7485, Trondheim, Norway; c University College Dublin, Belfield, Dublin 4, D04 N2E5, Ireland

**Keywords:** Classification, Hyperspectral, National vegetation classification (NVC), Plant species composition, Spectral, Vegetation

## Abstract

Remote sensing of vegetation provides important information for ecological applications and environmental assessments. The association between vegetation composition and structure with its spectral signal can most fully be assessed with hyperspectral data. Particularly field spectroscopy data can improve such understanding as the spectral data can be linked with the vegetation under consideration without the geographic registration uncertainties of aerial or satellite imagery. The data provided in this article contain field spectroscopy measurements from non-arable, grass-dominated objects on four farms in an intensively used agricultural landscape in the South-East of the UK. Detailed data on the plant species composition of the objects are also supplied with this article to support further analysis. Reuse potential includes linking the vegetation data with the spectral response using spectral unmixing techniques to map certain plant species or including the field spectroscopy data in a larger study with data from a wider area. This data article is related to the paper ‘Classifying grass-dominated habitats from remotely sensed data: the influence of spectral resolution, acquisition time and the vegetation classification system on accuracy and thematic resolution’ (Bradter et al., 2019) in which the ability to classify the recorded vegetation from the field spectroscopy data was analysed.

**Table:** Specifications Table

Subject	Ecology
Specific subject area	Vegetation and remote sensing
Type of data	Tables (Excel format)
How data were acquired	Field spectroscopy data were acquired with the spectroradiometers ASD Field Spec Pro and SVC HR-1024i, supplied and calibrated by the Natural Environment Research Council (NERC) Field Spectroscopy Facility (FSF). Compositional data of all vascular plant species were recorded in sample areas of 4 m^2^.
Data format	Raw, cleaned.
Parameters for data collection	Field spectroscopy data: at approximately monthly intervals May–August 2013 and in September 2012; in sunny conditions when no clouds were near the sun; 2–3 hours before and after solar noon. Vegetation data: in 4 m^2^ in the same periods as spectroscopy data were collected.
Description of data collection	Experienced vegetation surveyors identified areas with the same plant species composition and structure (vegetation category). For each vegetation category all vascular plant species and their percentage cover were recorded in 2–4 sample areas of 4 m^2^. Field spectroscopy data were collected at approximately monthly intervals in September 2012, August, July, June and May 2013 from as many vegetation categories as weather conditions allowed (849 spectra in total). For each vegetation spectrum, a reflectance spectrum from a Spectralon reference panel was recorded to calculate absolute reflectance (%). Field spectroscopy data: absolute reflectance (%) and metadata. Vegetation data: species name and its cover (%).
Data source location	UK; on four farms, S: 52.062798, W: −0.297005; N: 52.312038, E: 0.868366.
Data accessibility	Data are provided with this article.
Related research article	Data presented in this article is related to the research presented in U. Bradter, J. O'Connell, W.E. Kunin, C.W.H. Boffey, R.J. Ellis, T.G. Benton, Classifying grass-dominated habitats from remotely sensed data: the influence of spectral resolution, acquisition time and the vegetation classification system on accuracy and thematic resolution. Science of the Total Environment. Available online: 3 November 2019. https://doi.org/10.1016/j.scitotenv.2019.134584.

**Table:** 

**Value of the Data** • The datasets provide a direct link between vegetation composition and the spectral response of the vegetation which can advance ecological remote sensing applications. • Data is available for researchers to combine into larger, more comprehensive datasets or for additional analysis, such as deriving plant species within the mixed vegetation plots (e.g. through spectral unmixing) or studying changes of spectral vegetation reflectance over the growing season. • Field spectroscopy data is available with metadata of instrument settings, dates and times of data acquisition, thus enabling researchers to use the data for their own studies. • The field spectroscopy data contain a very high spectral information content due to the continuous narrow bands of the hyperspectral sensor. • The spectral data can be linked with the vegetation data from mostly narrow objects (e.g. field margins) without the geographic registration uncertainties of aerial or satellite imagery, which make remotely sensed vegetation assessment from narrow objects difficult.

## 1 Data

Remote sensing of vegetation is important for ecological applications and environmental assessment. Hyperspectral data have a high spectral information content enabling the differentiation of habitats at a high level of detail [1]. Hyperspectral data can be collected with hand-held instruments (field spectroscopy) thus enabling the direct linking of vegetation from narrow objects with their spectral reflectance without the geographic uncertainties of aerial and satellite imagery.

Vegetation survey data from grass-dominated non-arable habitats in an intensively used agricultural landscape in East Anglia, UK are presented together with field spectroscopy measurements. Field spectroscopy data (Tables 1–18) contain the absolute reflectance (%) measured over the wavelength range 400–2500 nm for each sample point. In September 2012, 132 spectra were recorded on the 7th (46 spectra, Table 1) 8th (25 spectra, Table 2), 13th (39 spectra, Table 3) and the 15th (22 spectra, Table 4). In May 2013, 195 spectra were recorded on the 1st (112 spectra, Table 5), 3rd (60 spectra, Table 6) and 27th (23 spectra, Table 7). In June 2013, 248 spectra were recorded on the 2nd (10 spectra, Table 8), 3rd (57 spectra, Table 9), 4th (43 spectra, Table 10), 6th (90 spectra, Table 11) and 7th (48 spectra, Table 12). 245 spectra were recorded in July 2013 on the 6th (59 spectra, Table 13), 9th (100 spectra, Table 14), 11th (34 spectra, Table 15), 17th (1 spectrum, Table 16) and 19th (50 spectra, Table 17). Field spectroscopy in August 2013 was only possible on the 31st (30 spectra, Table 18). The metadata for the field spectroscopy data recorded with the ASD Field Spec Pro contain the vegetation category, the date and time each spectrum was recorded and instrument settings (Table 19). For a detailed description of the ASD Field Spec Pro spectroradiometer, see Hatchell [2]. The metadata for the field spectroscopy data recorded with the SVC HR-1024i contain the vegetation category for each spectrum, the date and time each spectrum was recorded, the time difference between recording the spectrum of the target vegetation and of the reference panel and instrument settings (Table 20). For a detailed description of the SVC HR-1024i spectroradiometer explaining instrument settings, see Spectra Vista Corporation [3]. Start and end times for the recording of spectra per day and the number of spectra recorded per day are provided in Table 1.

**Table 1:** A summary of the datasets attached to this article containing the field spectroscopy measurements, with the spectroradiometer used (ASD: ASD Field Spec Pro; SVC: SVC HR-1024i), the date and time period during which spectra in each file were recorded and the number of spectra recorded per day. Times are British summer time (GMT + 1).

Table number	Instrument	Date	Start time	End time	No of spectra
Table 1	ASD	7th September 2012	11:19	15:14	46
Table 2	ASD	8th September 2012	11:13	15:22	25
Table 3	ASD	13th September 2012	11:30	13:06	39
Table 4	ASD	15th September 2012	14:30	15:11	22
Table 5	SVC	1st May 2013	11:10	15:03	112
Table 6	SVC	3rd May 2013	11:48	13:17	60
Table 7	SVC	27th May 2013	10:35	11:35	23
Table 8	SVC	2nd June 2013	10:01	10:39	10
Table 9	SVC	3rd June 2013	10:11	12:02	57
Table 10	SVC	4th June 2013	11:00	12:41	43
Table 11	SVC	6th June 2013	12:43	15:58	90
Table 12	SVC	7th June 2013	10:44	14:50	48
Table 13	SVC	6th July 2013	10:50	12:22	59
Table 14	SVC	9th July 2013	10:15	14:30	100
Table 15	SVC	11th July 2013	13:10	14:05	34
Table 16	SVC	17th July 2013	13:40	13:40	1
Table 17	SVC	19th July 2013	10:23	11:51	50
Table 18	SVC	31st August 2013	10:53	11:33	30

Percentage of ground covered by each vascular plant species recorded in sample areas of 4 m^2^ are supplied for vegetation categories recorded with the ASD Field Spec Pro (Table 22) and the SVC HR-1024i (Table 23). The data were used to study the spectral separability of vegetation categories depending on the month in which the spectra were recorded [4].

## 2 Experimental design, materials, and methods

**Fig. 1:**
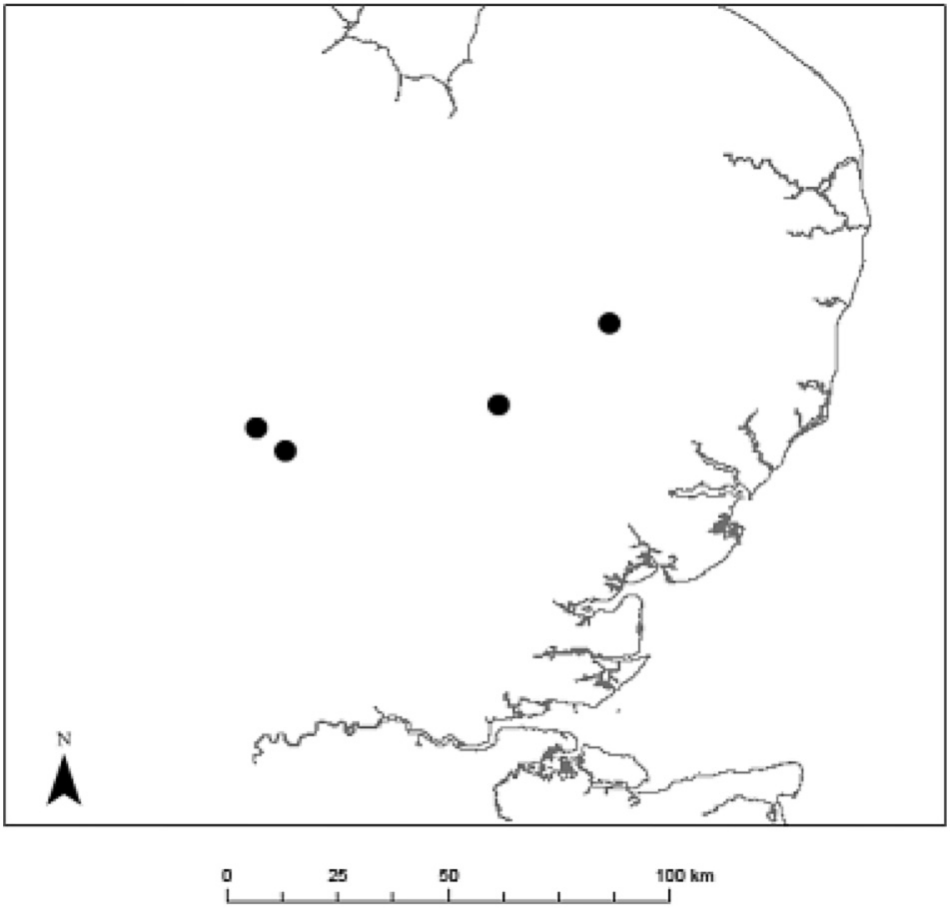
The location of the four field sites (black dots) in East Anglia, UK.

Experienced field surveyors mapped areas with relatively uniform species composition and structure (vegetation categories) on four farms in East Anglia, UK (Fig. 1). Several spatially separated objects were mapped per vegetation category (e.g. several field margins) if they were within ca. 10 min transfer time of each other. As blue sky conditions (no clouds near the sun and no haze) are desired for field spectroscopy, but are relatively rare in the UK, long transfer times between objects would have risked wasting rare blue sky conditions and longer travel distances between objects were therefore avoided (min/median/max distance between objects within a farm: 10/65/2850 m). To characterize the vegetation categories, all vascular plant species and their percentage cover were recorded in 2–4 sample areas of 4 m^2^ (depending on the number of objects per category). Most mapped areas were from narrow objects (e.g. field margins). Percentage cover was recorded from eye level height and can sum to more than 100% as foliage of different plant species frequently overlap.

Spectra were recorded during sunny conditions with no clouds near the sun. During the longest days of the year, data were collected within 3 hours of solar noon and during the shortest days within 2 hours of solar noon. The aim was to record spectra of all vegetation categories at approximately monthly intervals, but the days suitable for sampling and the number of spectra and vegetation categories recorded per day depended on weather conditions. Weather conditions were most restrictive in August 2013, when only 30 spectra could be collected late in the month. Per object, spectra were recorded ca. 9 m apart unless the object was very small when the distance was shortened accordingly. Spectra were recorded in 2012 with the ASD Field Spec Pro (Analytical Spectral Devices, Inc, Boulder, USA) using a 18° fore-optic and in 2013 with the SVC HR-1024i (Spectra Vista Corporation, New York, USA) using a 8° fore-optic. Spectra were recorded hand-held from a height of 1 m above the canopy, resulting in a field of view of 31.5 cm (ASD Field Spec Pro) and 13.9 cm (SVC HR-1041i). On average 21 spectra (median) per vegetation category per month were collected (min: 10; max: 78) from different locations within the vegetation category objects to cover the between-sample heterogeneity of vegetation.

Spectroradiometers were supplied by the Natural Environment Research Council (NERC) Field Spectroscopy Facility (FSF), UK. The instruments were calibrated and maintained by FSF. Instrument calibration included radiance and irradiance calibration and wavelength verification using standards calibrated by the National Physical Laboratory (see https://fsf.nerc.ac.uk/lab/), which ensured consistency between spectroradiometer measurements of the two instruments.

Absolute reflectance (%) of the target vegetation was calculated as: Target Spectra/Reference Panel Spectra * Reference Panel Calibration * 100, where the Target Spectra was from the vegetation category under consideration, the Reference Panel Spectra was a spectra taken of a Spectralon reference panel (Labsphere, North Sutton, USA) and the Reference Panel Calibration were the wavelength-specific calibration data of the reference panel. The calibration data were supplied by FSF from a calibration of the reference panel before the loan of each spectroradiometer. A spectra of the Spectralon reference panel was recorded immediately before or after the recording of a target spectra in order to calculate absolute reflectance using the same light conditions.
